# Tau accumulation in autosomal dominant Alzheimer’s disease: a longitudinal [^18^F]flortaucipir study

**DOI:** 10.1186/s13195-023-01234-5

**Published:** 2023-05-25

**Authors:** Antoinette O’Connor, David M. Cash, Teresa Poole, Pawel J. Markiewicz, Maggie R. Fraser, Ian B. Malone, Jieqing Jiao, Philip S. J. Weston, Shaney Flores, Russ Hornbeck, Eric McDade, Michael Schöll, Brian A. Gordon, Randall J. Bateman, Tammie L. S. Benzinger, Nick C. Fox

**Affiliations:** 1 Dementia Research Centre, UCL Queen Square Institute of Neurology, London, UK; 2 UK Dementia Research Institute at UCL, London, UK; 3 Department of Medical Statistics, London School of Hygiene & Tropical Medicine, London, UK; 4 Centre for Medical Image Computing, Medical Physics and Biomedical Engineering, UCL, London, UK; 5 Department of Radiology, Washington University in St. Louis, St. Louis, MO USA; 6 Department of Neurology, Washington University in St. Louis, St. Louis, MO USA; 7 Wallenberg Centre for Molecular and Translational Medicine, University of Gothenburg, Gothenburg, Sweden

## Abstract

Cortical tau accumulation is a key pathological event that partly defines Alzheimer’s disease (AD) onset and is associated with cognitive decline and future disease progression. However, an improved understanding of the timing and pattern of early tau deposition in AD and how this may be tracked in vivo is needed. Data from 59 participants involved in two longitudinal cohort studies of autosomal dominant AD (ADAD) were used to investigate whether tau PET can detect and track presymptomatic change; seven participants were symptomatic, and 52 were asymptomatic but at a 50% risk of carrying a pathogenic mutation. All had baseline flortaucipir (FTP) PET, MRI and clinical assessments; 26 individuals had more than one FTP PET scan. Standardised uptake value ratios (SUVRs) in prespecified regions of interest (ROIs) were obtained using inferior cerebellar grey matter as the reference region. We compared the changes in FTP SUVRs between presymptomatic carriers, symptomatic carriers and non-carriers, adjusting for age, sex and study site. We also investigated the relationship between regional FTP SUVRs and estimated years to/from symptom onset (EYO). Compared to both non-carriers and presymptomatic carriers, FTP SUVRs were significantly higher in symptomatic carriers in all ROIs tested (*p* < 0.001). There were no significant regional differences between presymptomatic carriers and non-carriers in FTP SUVRs, or their rates of change (*p* > 0.05), although increased FTP signal uptake was seen posteriorly in some individuals around the time of expected symptom onset. When we examined the relationship of FTP SUVR with respect to EYO, the earliest significant regional difference between mutation carriers and non-carriers was detected within the precuneus prior to estimated symptom onset in some cases. This study supports preliminary studies suggesting that presymptomatic tau tracer uptake is rare in ADAD. In cases where early uptake was seen, there was often a predilection for posterior regions (the precuneus and post-cingulate) as opposed to the medial temporal lobe, highlighting the importance of examining in vivo tau uptake beyond the confines of traditional Braak staging.

**Supplementary Information:**

The online version contains supplementary material available at 10.1186/s13195-023-01234-5.

## Introduction

Alzheimer’s disease (AD) is characterised by the presence of extracellular amyloid plaques and intracellular neurofibrillary tangles (NFT) composed of hyper-phosphorylated tau at post-mortem. The advent of tau tracers, which include the first generation tracer [^18^F]flortaucipir (FTP), as well as second generation tracers ([^18^F]MK6240, [^18^F]RO-948), [^18^F]PI2620, [^18^F]JNJ-311, [^18^F]GTP1) has enabled characterisation of tau regional distribution in vivo [1, 2]: FTP signal correlates with tau pathology at post-mortem [3, 4], distinguishes AD from healthy controls and other neurodegenerative conditions [5–7] and is associated with cognitive impairment, cortical hypometabolism, neurodegeneration and future disease progression [5, 8–11].

However, the ability of FTP to detect tau pathology in presymptomatic AD and the timing of when tau PET tracer signal starts to change in relation to symptom onset are still unclear. Some longitudinal studies in sporadic disease have shown increased rates of change of tau tracer uptake among cognitively unimpaired amyloid-beta positive groups [12, 13], while others have not [14, 15]. Understanding the early, often clinically silent, pathological changes underlying AD onset is critical to realising effective disease-modifying treatments: early intervention represents an opportunity to halt disease progression at a potentially more tractable disease stage when there is the most to save in terms of cognitive function.

Autosomal dominant Alzheimer’s disease (ADAD), an autosomal dominant condition caused by mutation in the genes *Presenilin1/2 (PSEN1/2)* and *amyloid precursor protein (APP)*, is a valuable model for examining preclinical AD [16]. The unique value of studying ADAD is that the young, reasonably consistent age at onset between family members, allied to the near 100% penetrance of mutations, means that studies of presymptomatic mutation carriers can prospectively characterise the sequence and timing of key pathological changes relative to symptom onset [17]. Additionally, the early age of typical disease onset precedes the age when age-related NFT deposition is found in older adults, thereby offering an opportunity to more closely track regional NFT changes related to AD [18] independent of age-related effects.

Cross-sectional studies in ADAD have shown very clear increases in tau PET signal in symptomatic disease, although evidence supporting presymptomatic tau signal change is inconsistent [19, 20]. A recent longitudinal study demonstrated increased rates of change in FTP signal in mutation carriers (symptomatic and asymptomatic); however, a separate comparison, restricted to presymptomatic carriers, was not performed [21]. There is an urgent need for an improved understanding of the preclinical trajectory of tau accumulation, especially as this hallmark protein is becoming a major target for neurodegenerative therapeutics, for example, in the recently launched DIAN-TU trial of an anti-tau antibody combined with an anti-amyloid antibody (NCT 05269394) [22]. The study presented here uses longitudinal tau PET data to investigate the rate and pattern of tau accumulation in presymptomatic and symptomatic ADAD cases, while also examining when changes in tau PET first discriminate between carriers and non-carriers.

## Materials and methods

### Study design and participants

Participants in this study were recruited from two sites: 19 participants from a longitudinal cohort study of ADAD at the Dementia Research Centre, University College London, and 43 from Washington University in St. Louis as part of the Dominantly Inherited Alzheimer’s Network (DIAN) observational study [19, 23]. All visits were performed between March 2015 and July 2019. The eligibility criteria were (i) being aged 18 or over and either (ii) a clinical diagnosis of ADAD or (iii) being asymptomatic but “at-risk” of developing ADAD, i.e. having an ADAD-affected parent and thus being at a 50% risk of inheriting a pathogenic mutation and thereby of developing symptoms at a similar age to their affected parent. The exclusion criteria were co-existing neurological or psychiatric diseases that interfered with cognition or contraindications to PET or MRI. At enrolment, seven participants were symptomatic, with pathogenic mutations in PSEN1/2 or APP genes; 55 individuals were asymptomatic “at-risk”—this group contained a mix of presymptomatic mutation carriers and non-carriers.

One asymptomatic participant was more than 10 years beyond the expected age at onset and had an inconsistent cognitive profile on neuropsychology testing and was therefore excluded from the dataset. The study was approved by the local Research Ethics Committee at each site, and written informed consent was obtained from participants or their caregivers.

### Procedures

ADAD mutation status was determined using Sanger sequencing at both sites; results for all participants were provided only to statisticians, ensuring the blinding of participants, clinicians and those performing imaging analysis.

All participants identified an informant who was interviewed separately for a collateral history. Estimated years from symptom onset (EYO) were calculated by subtracting the age at which the participant’s affected parent first developed progressive cognitive symptoms from the participant’s age at assessment.

Individuals were defined as symptomatic if (i) cognitive decline was reported by participant and/or their informant, (ii) the clinical impression was that the participant was experiencing progressive decline, (iii) the global Clinical Dementia Rating (CDR®) scale was > 0 [24] and (iv) an alternative cause of cognitive decline was not identified.

### Image acquisition

PET scanning was performed at an approximate target time interval of 2 years. Both sites used Siemens Biograph PET/CT scanners. Due to the differences in regulatory requirements, the amount of FTP dose injected depended on the site (141 ± 17 MBq for UCL, 322 ± 32 MBq for Washington University).

For all PET scans, a low-dose CT scan was performed for attenuation correction. Images were acquired at 80–100 min post-injection. Reconstruction parameters were harmonised across sites, using an ordered subset expectation maximisation algorithm with 3 iterations and 24 subsets (21 for DIAN). The use of the same scanner at both sites and the similarity of reconstruction parameters meant that a harmonising smoothing kernel did not need to be applied.

Structural MRI at each site was acquired on a Siemens Prisma scanner with a 3D magnetisation-prepared rapid gradient echo (MPRAGE) T1-weighted scan. A subset of individuals (*N* = 26) returned for one (*N* = 23) or two (*N* = 3) repeat visits with tau PET and MRI.

### Image processing

Within each session, all PET frames representing 80 to 100 min post-injection were motion corrected and averaged to form mean non-attenuation corrected (NAC) and attenuation corrected (AC) PET images using the spm_realign function in the SPM12 toolbox. MRI data was processed using the Geodesic Information Flow (GIF) algorithm which generates a subject-specific whole brain and cerebellar parcellation [25]. The cerebellar parcellation, based on the Spatially Unbiased Infratentorial Template for the cerebellum (SUIT) template [26], was used to generate an inferior cerebellar grey reference region, similar to the one previously outlined [27]. Rigid registration of MRI to the mean NAC PET data was performed using the NiftyReg package [28] in order to transfer the brain and cerebellar parcellations into native PET space. Standardised uptake value ratio (SUVR) on the AC PET images was created using the inferior cerebellar GM region of interest as the reference region by which FTP uptake in any given target region was divided. Five target regions were chosen, based on sporadic AD and ADAD literature [19, 29–31]: (1) the entorhinal cortex, which approximates to a Braak I/II stage; (2) a temporal lobe meta-ROI that approximates to a Braak III/IV; and (3) a global composite ROI that represents the areas found in Braak V/VI [13, 29]. This more global composite ROI does not include the posterior cingulate and the precuneus; these regions were assessed separately given their susceptibility to tau accumulation and early atrophy in ADAD [19, 30].

Longitudinal processing followed a similar pipeline to the cross-sectional data (Supplementary Fig. 1). First, the T1-weighted MR scans were registered together using the pairwise longitudinal registration function in SPM12 to generate a midpoint image [32]. Parcellation using GIF was then performed on the resulting midpoint image. The resulting deformation fields from the longitudinal registration were composed of the rigid body transformations from the co-registration performed above in order to map consistent anatomical brain regions from midpoint space into each time point’s native PET space. Image processing was performed blind to participants’ mutation status and clinical diagnosis. There were two individuals whose tau PET scans did not pass quality control due to motion artefact and they were excluded from the subsequent analyses, reducing the number of individuals included in the study to 59 (7 symptomatic and 52 asymptomatic) (flow chart on participant exclusions Supplementary Fig. 2).

### Statistical analysis

Baseline (first visit) summary statistics were calculated for our three groups (symptomatic mutation carriers, presymptomatic carriers, non-carriers), and box plots of baseline FTP SUVRs were produced for each of the five ROIs showing the three groups. All other plots and analyses used data from all visits. Longitudinal plots of FTP SUVR against EYO were produced for each ROI, identifying the three groups; these plots were then redrawn using actual years to/from onset (AYO) instead of EYO, for these participants who had become symptomatic. Differences in FTP SUVR between the groups (non-carriers (*n* = 23), presymptomatic carriers (*n* = 29), symptomatic carriers (*n* = 7)), adjusted for age at visit, sex and study site, were estimated for each ROI using linear mixed effect models that included random intercepts at both family and individual levels, in each case with different variances by group where appropriate, and additionally allowing for the variances of the model residuals to differ by group; pairwise comparisons were only carried out when a joint test across all groups showed evidence of a difference. Group comparison models that also included random slopes for age at visit, separately by group, did not converge. For the 26 study participants with more than one visit, the rate of change in FTP SUVR was calculated between the first and last visit (three individuals had three visits). Differences in the rate of change for each ROI between non-carriers (*n* = 9) and presymptomatic carriers (*n* = 15) were estimated using linear regression models adjusting for age at the mid-point between first and last visit, sex and study site; robust standard errors were used for the posterior cingulate and precuneus analyses due to heteroskedastistic residuals. There was no evidence to allow for clustering by family. The rate of change models was then repeated adding baseline FTP SUVR as an additional covariate. Rates of change analyses were not performed for the symptomatic group due to small numbers (*n* = 2 with more than one visit).

The relationship between FTP SUVR and EYO was modelled using a linear mixed effect model for each ROI (the number of participants included in these analyses was as given above for the FTP SUVR group comparison models). The fixed effects were age at visit, gender, study site, mutation status (carrier vs non-carrier), EYO, an interaction between carrier status and EYO and, for mutation carriers only, a quadratic term for EYO. A random intercept was included for individuals from the same family and mutation status; in addition, residual variances were allowed to differ by mutation carrier status. Models that additionally included a (correlated) random slope for EYO, separately for mutation carriers and non-carriers, either did not converge or converged with the estimated correlation between slope and intercept being unity; we therefore did not include random slopes. The estimated mean FTP SUVRs (and 95% confidence intervals) for mutation carriers and non-carriers were plotted against EYO, standardised to a population with equal numbers of males/females, equal representation of the two study sites and aged 38.9 years (the mean baseline age of our participants). In order to estimate the time point at which there was evidence of divergence by mutation carrier status, we calculated the estimated difference in mean FTP SUVR between carriers and non-carriers, after adjusting for age, sex and study site, for integer values of EYO between − 20 and 10 years. The point when this estimated difference was statistically significantly different from zero (*p* ≤ 0·05) was interpreted cautiously as an indication of when (by EYO point) there was evidence that the estimated trajectory of FTP SUVR for mutation carriers diverged from non-carriers. In sensitivity analyses, we re-fitted these trajectory models omitting outlier observations (those with standardised residuals with a magnitude greater than 2).

No correction to the statistical significance level was made for multiple comparisons [33]. All analyses were performed using Stata v16.

### Data availability

Data are available upon reasonable request from qualified investigators, adhering to ethical guidelines (https://dian.wustl.edu/our-research/for-investigators/dian-observational-study-investigator-resources/).

## Results

### Participant demographics

Baseline demographic and clinical characteristics are presented in Table 1; presymptomatic mutation carriers were reasonably well-matched with non-carriers in terms of age and were, as expected, younger than symptomatic carriers. The mean EYO for presymptomatic mutation carriers was −13.6 years.

**Table 1:** Baseline characteristics

	**Non-carrier**	**PMC**	**SMC**
***N***	23	29	7
**Sex, *n* (%)**			
**Men**	16 (70%)	16 (55%)	5 (71%)
**Women**	7 (30%)	13 (45%)	2 (29%)
**Age, years (mean (SD))**	40.9 (12.4)	34.6 (7.0)	49.8 (9.5)
**EYO, years (mean (SD))**	N/A	-13.6 (7.5)	2.5 (3.9)
**MMSE (median [IQR])**	30 [29, 30]	30 [29, 30]	27 [20, 29]
**CDR Global (median [IQR])**	0 [0, 0]	0 [0, 0]	0.5 [0.5, 1]
**Tau PET follow-up interval**^**a**^**, years (mean, (SD), number with more than one visit)**	2.1 (0.8) *n* = 9	2.4 (1.0) *n* = 15	2.8 (1.9) *n* = 2

*PMC* presymptomatic mutation carrier, *SMC* symptomatic mutation carrier

^a^For participants with more than one visit

#### Tau PET change in symptomatic ADAD

**Fig. 1:**
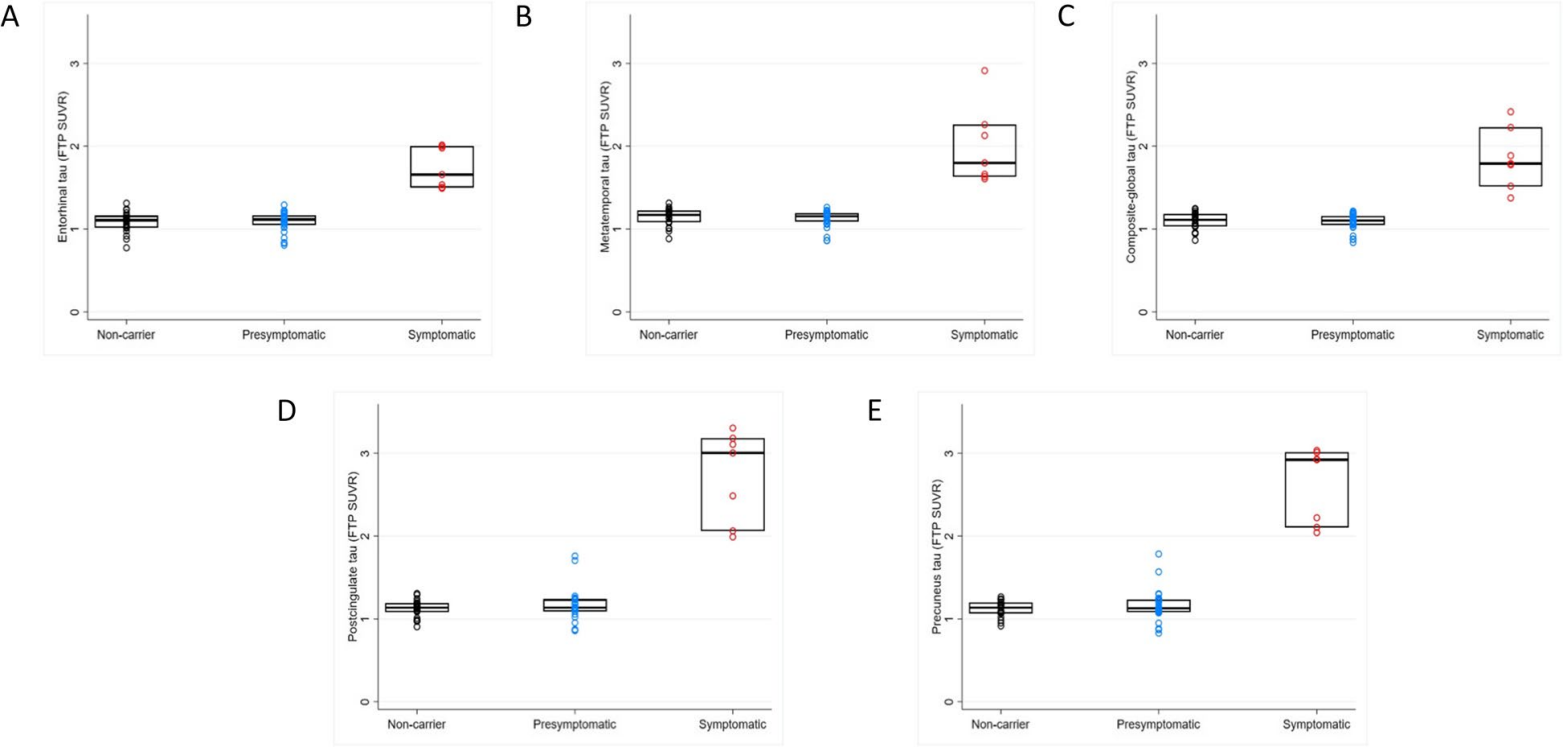
Box plots for observed baseline FTP SUVR values across the three groups. The observed FTP values in all 5 ROIs (**A** entorhinal, **B** meta temporal, **C** composite global, **D** posterior cingulate, **E** precuneus) at baseline are shown. Mutation carriers have been divided into those who are symptomatic and those who are presymptomatic. Boxes show the median and first and third quartiles. Dots represent individual observations

Adjusting for age at visit, sex and study site, FTP SUVRs in all 5 ROIs were significantly greater in symptomatic mutation carriers compared to both non-carriers and presymptomatic carriers (observed baseline data shown in Fig. 1; all *p*-values < 0.001 for adjusted comparisons with symptomatic mutation carriers, as shown in Supplementary Table 1). The greatest magnitude of difference between non-carriers and symptomatic mutation carriers was seen within the precuneus and posterior cingulate gyrus regions.

**Fig. 2:**
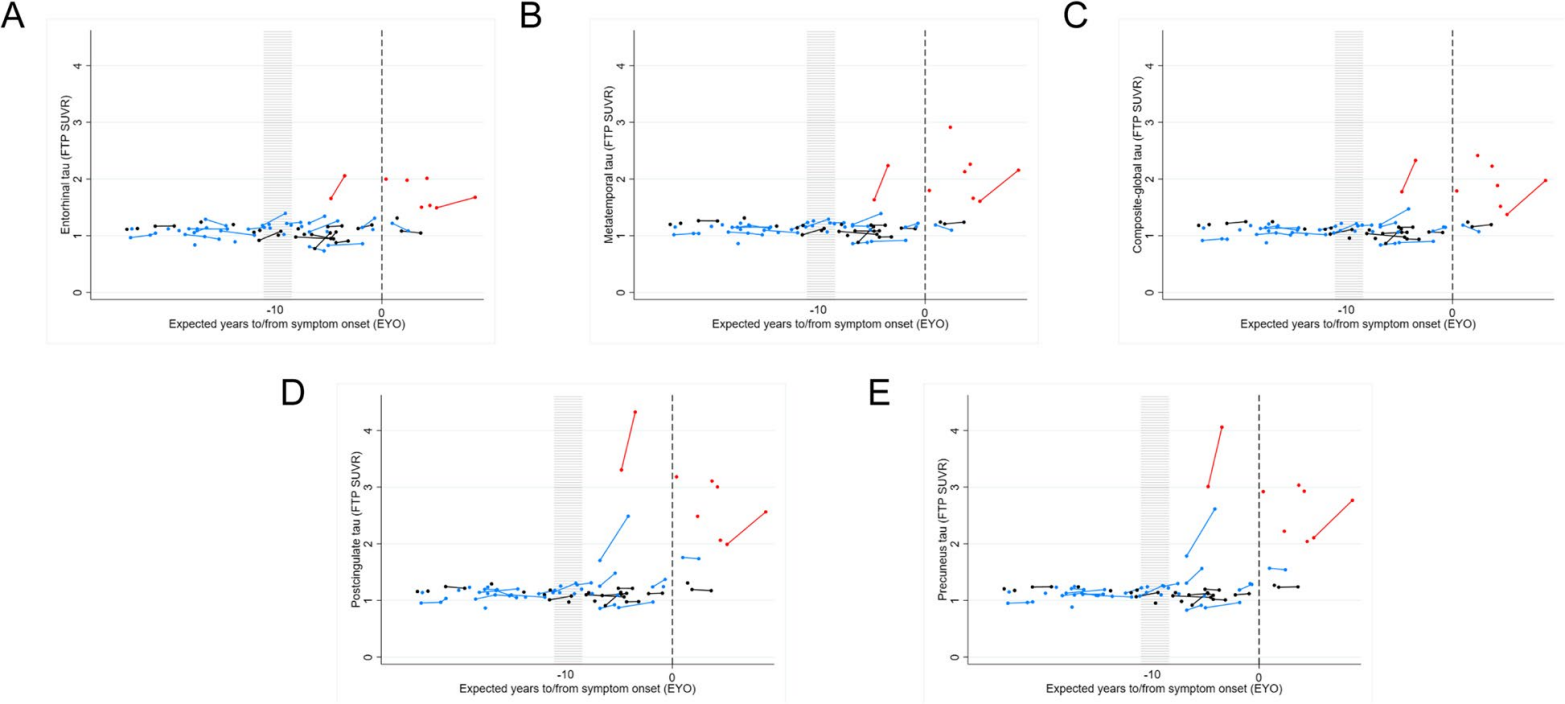
FTP signal uptake against EYO. Observed values of FTP uptake in all 5 ROIs (**A** entorhinal, **B** meta temporal, **C** composite global, **D** posterior cingulate, **E** precuneus) against EYO. Symptomatic mutation carriers are represented in red, presymptomatic carriers in blue, and non-carriers in black. Those measurements that belong to the same individual are connected by a line. To maintain blinding of mutation status, the values of the *x*-axis for all EYO plots have been removed except for EYO = 0, indicated by a broken line, plus an approximate indication of EYO =  −10; additionally, four asymptomatic individuals with an EYO > 10 are also not shown (but these data were included in all analyses)

The small number of symptomatic individuals with more than one visit (*N* = 2 with longitudinal imaging) precluded formal statistical comparisons of rates of change in symptomatic carriers against both non-carriers and presymptomatic carriers. However, spaghetti plots demonstrated increases in FTP signal over the course of the scanning interval for these two symptomatic individuals (Fig. 2), with very high rates of change seen especially in the precuneus and posterior cingulate (0.14 and 0.72 SUVR/year), although there was also clear change within entorhinal and meta-temporal regions (0.13 and 0.41 SUVR/year). When data was replotted using AYO, where known, very marked increases were seen for one of these individuals at the point of symptom onset (Supplementary Fig. 3).

#### Tau PET change in presymptomatic ADAD

Adjusting for age at visit, sex and study site, there were no statistically significant differences in FTP SUVRs between presymptomatic carriers and non-carriers (all *p*-values ≥ 0.12; Supplementary Table 1a; all the parameter estimates from this model, with 95% confidence intervals, are reported in Supplementary Table 1b). Similarly, statistical analyses did not find any significant differences in age, sex and study site-adjusted rates of change in FTP SUVR between presymptomatic carriers and non-carriers in the ROIs investigated (*p*-values ≥ 0.08; Supplementary Table 2a; all the parameter estimates from this model, with 95% confidence intervals, are reported in Supplementary Table 2b). There was no meaningful change to the results when baseline FTP SUVR was added to the rate of change models as an additional covariate and for no ROI was the estimated adjusted association between FTP SUVR and rate of change statistically significant.

**Fig. 3:**
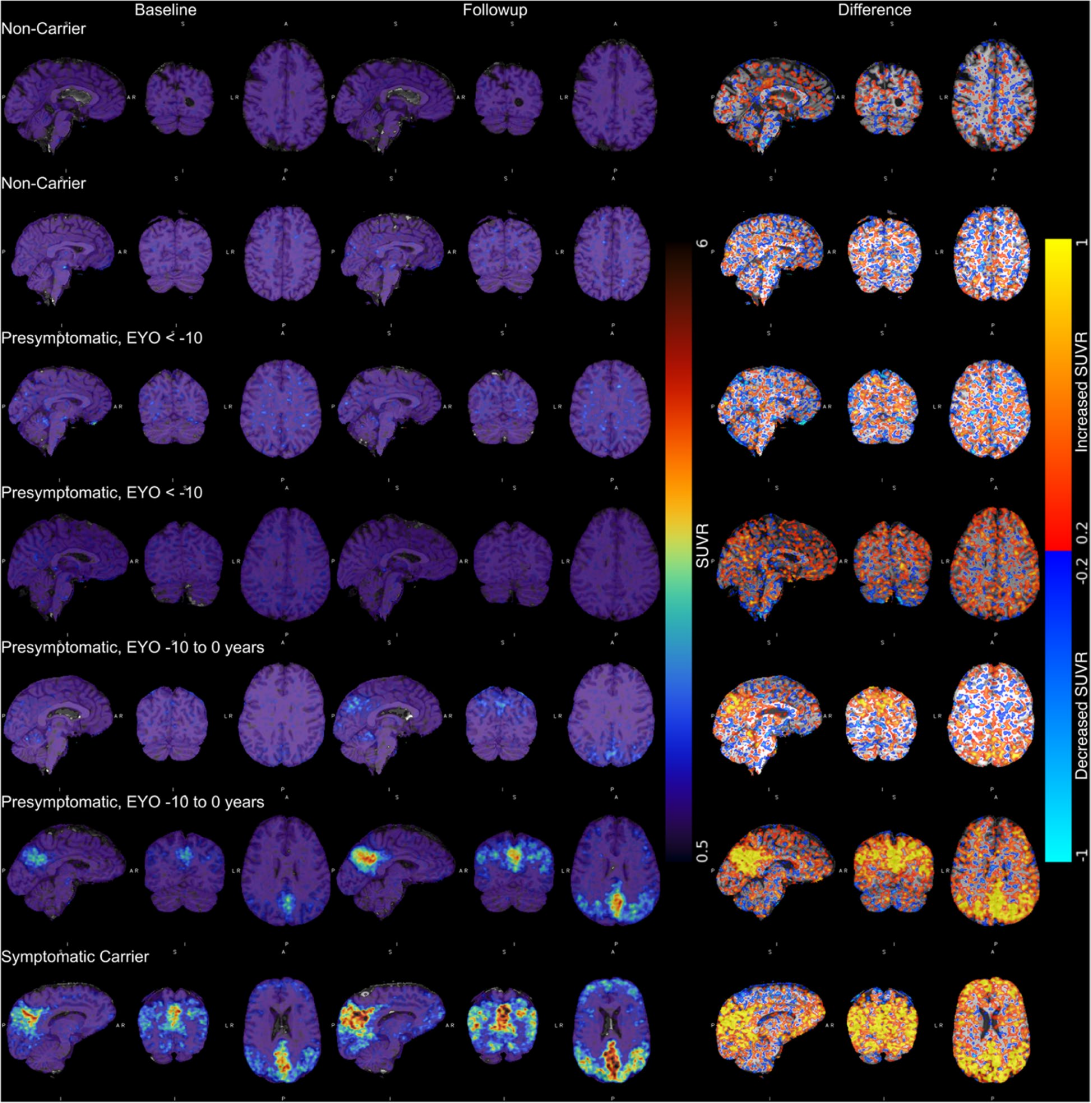
Longitudinal tau PET images from a selection of ADAD family members. The images have been smoothed with a Gaussian kernel (full-width at half maximum = 3 mm) in order to provide easier visual interpretation. The first two columns represent three cardinal slice views on the precuneus at baseline and follow-up time points. All of these SUVR images are mapped to the same colour scale. The third column represents the difference image between the two time points, with warm colours showing an increase in tau deposition and cool colours representing a decrease

At an individual level, spaghetti plots demonstrated a heterogenous pattern of FTP uptake change among presymptomatic carriers, even within the same EYO range (Fig. 2). However, it is interesting to note that a small cluster of presymptomatic carriers, nearing symptom onset, showed very striking increases in observed FTP signal; these changes were most clearly seen in the precuneus and posterior cingulate region (Fig. 2, tau PET images for illustrative cases displayed in Fig. 3).

#### Pattern and timing of tau PET signal change

Visual inspection of PET images of symptomatic mutation carriers revealed prominent and extensive FTP uptake across the neocortex (illustrative cases displayed in Fig. 3). In contrast, very little uptake and signal change was seen in non-carriers, while longitudinal imaging of some presymptomatic mutation carriers who were approaching symptom onset did show increasing FTP uptake, most notably in extra-temporal, posterior regions (Fig. 3).

The earliest significant difference in FTP SUVR values across the 5 ROIs studied was within the precuneus, with significant adjusted differences between mutation carriers and non-carriers being detected from up to 10 years prior to estimated (parental) symptom onset; however, this number needs to be treated cautiously given the small number of individuals involved and the non-linear nature of the changes (Supplementary Fig. 4, Supplementary Table 3a; all the parameter estimates from this model, with 95% confidence intervals, are reported in Supplementary Table 3b).

Significant adjusted differences in FTP SUVRs were also detected between non-carriers and carriers in the other ROIs studied: from an estimated 7 years (posterior cingulate and entorhinal) and 6 years (meta-temporal and global composite) prior to symptom onset (Supplementary Fig. 4, which also includes sensitivity analyses).

## Discussion

This longitudinal tau-PET study of ADAD demonstrated significant group differences in FTP values between symptomatic carriers and both presymptomatic carriers and non-carriers. However, neither these FTP SUVRs nor their estimated rates of change detected increased signal in presymptomatic carriers compared to non-carriers. That said, some presymptomatic carriers approaching estimated symptom onset did display very clear longitudinal increases in FTP signal, most prominently in posterior neocortical regions; this is consistent with FTP deposition within the precuneus being among the first regions to discriminate between mutation carriers and non-carriers, although the exact timing of these changes must be treated cautiously given our small size, distribution of the data, the non-linear nature of the very rapid changes and under-sampling of those approaching symptom onset.

The suggestion of relatively early FTP accumulation in the precuneus and posterior cingulate gyrus suggests that these extra-temporal posterior regions may be particularly susceptible to early tau accumulation in ADAD. This is perhaps unsurprising given that these regions are also among the earliest regions to show increased rates of atrophy in ADAD [30, 34] and is consistent with tau burden and atrophy being spatio-temporally correlated [35, 36]. However, the finding that tau accumulation can be detected in these posterior cortical regions at around the same time, or potentially even before, entorhinal change is interesting given the traditional Braak staging of tau spread described in *sporadic* AD, and raises the possibility that there are differences in tau deposition pattern between ADAD and sporadic AD [37]. This difference may be attributable to AD-dependent or independent mechanisms, such as age-related tau accumulation in medial temporal lobes contributing to signal increases in late-onset AD [38]. Alternatively, the lack of early entorhinal tau PET signal may be partly attributable to AD- and age-independent methodological reasons: the low spatial resolution of PET complicates the accurate quantification of the SUVRs in small volume regions like the entorhinal cortex, whereas post-mortem histological examination is better suited to the examination of this thin cortical structure. This possible discrepancy may also be partly due to pathological differences between sporadic and ADAD; tau pathology in ADAD has a predilection for posterior regions, while temporal tau levels tend to be earlier and higher in sporadic disease [19, 39]. That said, early FTP signal in the precuneus is not a feature exclusive to ADAD: this region has demonstrated high FTP signal in sporadic EOAD and some studies of prodromal AD [40–43]. The precuneus and posterior cingulate gyrus correspond to key hubs in the default mode network, which is known to be particularly susceptible to tau pathology [44, 45]. In addition, data-driven modelling of tau spread has revealed that tau deposition in just under 20% of sporadic AD cases tends to spare the medial temporal lobe and is instead characterised by early tracer build-up in the precuneus [46]. Taken together, these findings highlight the value of examining in vivo tau accumulation in regions beyond the confines of traditional Braak staging.

The finding that tau PET SUVRs were increased in symptomatic carriers compared to non-carriers is consistent with prior studies in ADAD [19, 21]. Notably, the two symptomatic cases in this study had very high rates of change of FTP (0.11 and 0.56 SUVR/year); these values are greater than the ~ 0.05 SUVR/year change in FTP reported in previous longitudinal studies of sporadic AD [13, 15]. Similarly, high rates of FTP signal change have also been reported in a separate longitudinal study involving symptomatic *PSEN1* E280A mutation carriers. The high signal intensity seen in symptomatic ADAD may be partly attributable to the pathobiology of dominantly inherited AD: neuropathology work has shown a higher density of neurofibrillary tangles in ADAD compared to sporadic AD [47, 48]. However, high rates may also be somewhat due to an age effect; greater levels of FTP signal, and higher accumulation rates, have been found in early (onset before 65 years of age) compared to late-onset sporadic AD [15, 49, 50]. This suggests that neurodegenerative processes, and in particular the accumulation of tau, may be more aggressive in earlier onset and in (at least some) familial forms of AD.

This study did not detect a difference in either FTP SUVR measures or rates of change in FTP SUVR between presymptomatic carriers and non-carriers. These results are broadly in line with a previous tau study that showed that absolute SUVR levels did not differ between presymptomatic carriers and non-carriers, although another study did show a significant increase in tau signal within the parahippocampal gyrus in presymptomatic compared to non-carriers [19, 20]. However, a subsequent longitudinal study from the same group did not detect a significant difference between mutation carriers and non-carriers in rates of change within either inferior temporal or entorhinal regions [21]. Taken together with our results, these findings suggest that significant increases in tau PET signal are not consistently detected in ADAD prior to symptom onset. Future larger studies that contain greater numbers of mutation carriers approaching symptom onset are needed to clarify when tau PET signal change begins in ADAD.

The absence of significant change in our presymptomatic group may also be partly due to under-sampling of those close to symptom onset (average EYO = − 13.6 years), as presymptomatic signal change (when seen) occurred in individuals nearing symptom onset. The ability of longitudinal tau PET to detect signal increases in preclinical sporadic AD is also somewhat unclear as studies have not consistently detected significantly increased FTP signal in cognitively normal, amyloid-positive compared to amyloid-negative groups [13–15, 51]. As in our study, the absence of group differences may be partly due to an over-representation of individuals far from symptom onset, as significantly higher longitudinal FTP accumulation rates, compared to amyloid-negative cases, are not found in all amyloid-positive individuals but only among a subset with very high levels of amyloid accumulation (> 68 Centiloids on amyloid PET) [12].

This study has a number of limitations including the small sample size and the use of EYO as a proxy measure of symptom onset. EYO, while a reasonable indicator of future symptom onset [17], is not without error due both to variability in age at onset between family members and to imprecision in determining the time of cognitive decline in a preceding, now often deceased, generation [52]. Therefore, to have a greater understanding of the exact timing of tau changes in ADAD, future studies should ideally investigate the distribution of tau SUVRs relative to actual, as opposed to estimated, symptom onset. Additionally, it will also be important for future studies to characterise other drivers of heterogeneity, in particular the impact of different lifestyle factors and genetic modifiers on the pathobiology of disease. For example, homozygosity of the *APOE3* Christchurch mutation has already been suggested to confer resistance to tau pathology in ADAD: a *PSEN1* E280A amyloid-positive mutation carrier had both limited and atypical distribution of tau signal on PET scanning despite being over 30 years beyond the median age of onset of mild cognitive impairment [53].

It is also important to consider the findings of this study in the context of the inherent limitations of the techniques used. Firstly, due to the relatively recent availability of tau-PET, and in particular the limited number of longitudinal tau PET studies, consensus has yet to emerge on the optimal post-processing method for longitudinal analysis, although this is a growing focus of research interest [54]. In particular, debate is ongoing about the use of partial volume correction, the most appropriate reference regions for SUVR analysis and ideal modelling methods [55, 56]. Secondly, as mentioned, the low spatial resolution of PET may complicate the accurate quantification of the SUVRs in small-volume regions like the entorhinal cortex. Thirdly, off-target tracer binding, which is seen in the choroid plexus and basal ganglia, can obscure accurate quantification of SUVRs within nearby ROIs such as the medial temporal lobe—a region which is outperformed by both the lateral temporal and global ROIs in terms of test–retest repeatability [55, 57, 58]. A final important limitation of longitudinal FTP studies is the kinetics of this tracer, which does not reach a steady state even at 130 min post-injection [59]. This is particularly problematic for longitudinal SUVR analysis as minor differences in the timing of scanning could potentially compromise the longitudinal consistency of intra-individual PET sessions.

## Conclusion

This study demonstrates that tau PET tracer uptake is usually not detected presymptomatically in ADAD, although increases may be observed in regions (such as the precuneus) in mutation carriers near symptom onset. Therefore, it will be important for future studies investigating the timing and sequence of tau spread in AD to include the study of uptake in this region.

This study also shows that symptomatic carriers have very high FTP signal, along with dramatic increases in tau deposition over quite short intervals. This suggests that the tau accumulation (at least as measured by tau PET) may be especially aggressive in ADAD and that because tau pathology accumulation is so linked with clinical onset, drugs that can prevent, stabilise or reverse tau pathology may have clinical benefits in AD.

## Supplementary Information

**Additional file 1: Supplementary Figure 1.** Schematic of longitudinal PET processing pipeline. **Supplementary Figure 2.** Flowchart of included and excluded cases. **Supplementary Figure 3.** FTP signal uptake against EYO/AYO. **Supplementary Figure 4.** Trajectory of FTP SUVR against estimated years to/from symptom onset. **Supplementary Table 1a.** Estimated differences* in mean FTP SUVR uptake (and 95% CI) across regions of interest, after adjusting for age, sex and study site. **Supplementary Table 1b.** Estimated covariate coefficients from the FTP SUVR group comparison models reported in Supplementary Table 1a. **Supplementary Table 2a.** Estimated difference* in mean rates of change of FTP SUVR uptake (SUVR/year) (and 95% CI) across regions of interest, after adjusting for age, sex and study site. **Supplementary Table 2b.** Estimated covariate coefficients from the FTP SUVR rates of change group comparison models reported in Supplementary Table 2a. **Supplementary Table 3a.** Ordering of outcomes by estimated years to symptom onset (EYO) point at which a significant difference in estimated mean FTP uptake was identified between mutation carriers (MC) and noncarriers (NC), after adjusting for age at visit, sex and study site. **Supplementary Table 3b.** Estimated covariate coefficients from from the models reported in Supplementary Table 3a, which were used to obtain the trajectories shown in Supplementary Figure 3.

## Data Availability

Supplementary material provided. Data are available upon reasonable request from qualified investigators, adhering to ethical guidelines.

## Abbreviations

AD: Alzheimer’s disease
ADAD: Autosomal dominant Alzheimer’s disease
APP: Amyloid precursor protein
CDR: Clinical Dementia Rating scale
DIAN: Dominantly Inherited Alzheimer’s Network
EYO: Estimated years to/from symptom onset
FTP: Flortaucipir
GIF: Geodesic Information Flow
NAC: Non-attenuation corrected
NFT: Neurofibrillary tangles
PET: Positron emission tomography
PSEN: Presenilin
ROI: Regions of interest
SUIT: Spatially Unbiased Infratentorial Template
SUVR: Standardised uptake value ratio
UCL: University College London
