# Supplementary material for: Tau accumulation in autosomal dominant Alzheimer’s disease: a longitudinal [18F]flortaucipir study
Source: Alzheimers Res Ther. 2023 May 25;15:99. doi: 10.1186/s13195-023-01234-5 (PMC10210376; doi:10.1186/s13195-023-01234-5)
Supplement: Supplementary file 1 — Additional file 1: Supplementary Figure 1. Schematic of longitudinal PET processing pipeline. Supplementary Figure 2. Flowchart of included and excluded cases. Supplementary Figure 3. FTP signal uptake against EYO/AYO. Supplementary Figure 4. Trajectory of FTP SUVR against estimated years to/from symptom onset. Supplementary Table 1a. Estimated differences* in mean FTP SUVR uptake (and 95% CI) across regions of interest, after adjusting for age, sex and study site. Supplementary Table 1b. Estimated covariate coefficients from the FTP SUVR group comparison models reported in Supplementary Table 1a. Supplementary Table 2a. Estimated difference* in mean rates of change of FTP SUVR uptake (SUVR/year) (and 95% CI) across regions of interest, after adjusting for age, sex and study site. Supplementary Table 2b. Estimated covariate coefficients from the FTP SUVR rates of change group comparison models reported in Supplementary Table 2a. Supplementary Table 3a. Ordering of outcomes by estimated years to symptom onset (EYO) point at which a significant difference in estimated mean FTP uptake was identified between mutation carriers (MC) and noncarriers (NC), after adjusting for age at visit, sex and study site. Supplementary Table 3b. Estimated covariate coefficients from from the models reported in Supplementary Table 3a, which were used to obtain the trajectories shown in Supplementary Figure 3. [file 13195_2023_1234_MOESM1_ESM.docx]

**Supplementary Material**


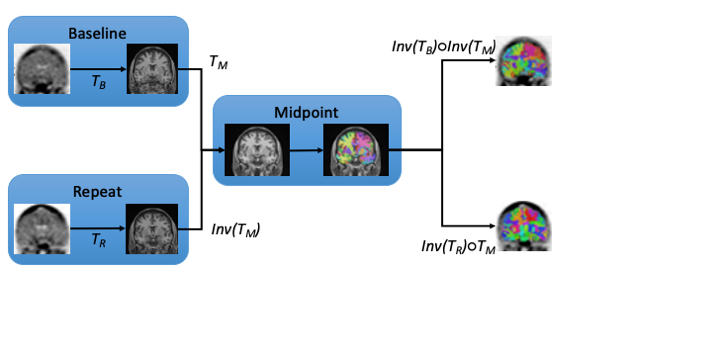


Supplementary Figure 1 Schematic of longitudinal PET processing pipeline.

T_B_ and T_R_ represent the PET to MR rigid transformations for the baseline and repeat timepoints. The baseline and follow-up T1-weighted scans were non-linearly registered using the Statistical Parametric Mapping algorithm that is optimised for longitudinal change. The longitudinal registration produces a T1 in a midpoint space between the two timepoints. The midpoint T1 was then parcellated using GIF. Transformations from the midpoint T1 to the individual MR timepoints (T_M_ ) were composed with the individual PET-MR rigid registrations to produce a mapping from the midpoint T1 directly into the native PET ensuring consistent ROIs were being used for the SUVR analysis across timepoints. Partial volume correction was not performed.

Supplementary Figure 2 Flowchart of included and excluded cases.


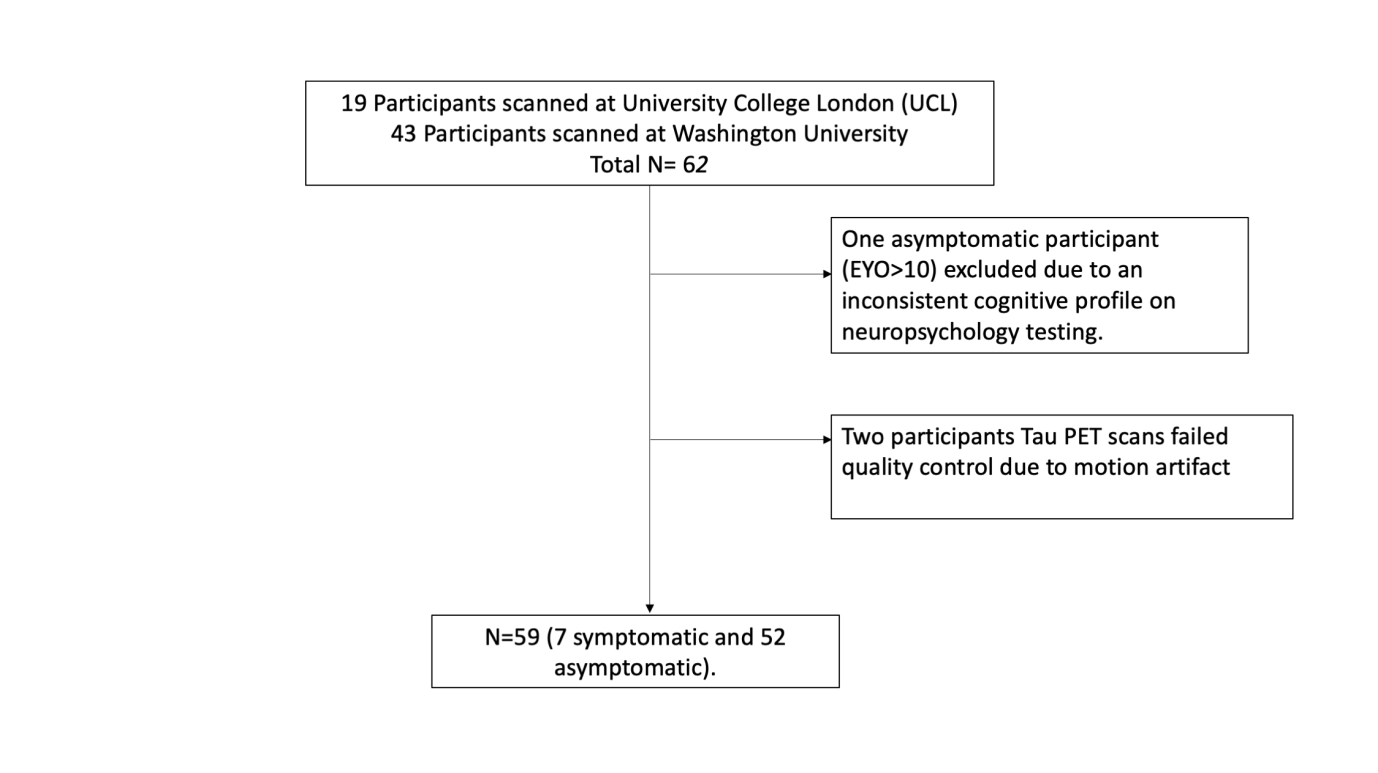


**Supplementary Figure 3**

Supplementary Figure 3: FTP signal uptake against EYO/AYO. Observed values of FTP uptake in all 5 ROIs (A = Entorhinal, B= Meta temporal, C = Composite Global, D = Posterior Cingulate, E = Precuneus) against EYO/AYO. Symptomatic mutation carriers are represented in red, presymptomatic carriers in blue, and non-carriers in black. Those measurements that belong to the same individual are connected by a line. To maintain blinding of mutation status, the values of the x-axis for all EYO plots have been removed except for EYO/AYO = 0, indicated by broken line, plus an approximate indication of EYO = -10; additionally four asymptomatic individuals with an EYO>10 are also not shown (but these data were included in all analyses).

**Supplementary Figure 4**

**Trajectory of FTP SUVR against estimated years to/from symptom onset.** Graphs compare mutation carriers (red) and non-carriers (blue) for estimated mean FTP uptake, with the x-axes representing estimated time to/from onset of progressive cognitive symptoms (EYO). Graphs are shown for each ROI (A = Entorhinal, B= Meta temporal, C = Composite Global, D = Posterior Cingulate, E = Precuneus). The plots presented are standardized to a population with equal numbers of males/females, equal representation of the two study sites, and aged 38.9 years (the mean baseline age of our participants). Dotted lines indicate 95% confidence intervals. Significant adjusted differences in FTP SUVRs were also detected between non-carriers and carriers in the all ROIs studied: from an estimated 10 years (precuneus), 7 years (posterior cingulate and entorhinal) and 6 years (meta-temporal and global composite) prior to estimated symptom onset. In sensitivity analyses where models were refitted omitting outlier values (3 outliers for entorhinal and posterior cingulate, 2 for global composite and precuneus, 1 otherwise), the ordering of first detectable group difference was similar i.e. first significant for precuneus (EYO = -7 years), posterior cingulate (EYO = -7 years), then meta-temporal (EYO = -6 years), global composite (EYO = -6 years) and finally entorhinal region (EYO = -5 years).

**Supplementary Table 1a: Estimated differences* in mean FTP SUVR uptake (and 95% CI) across regions of interest, after adjusting for age, sex and study site**

| **Region** | **Joint test of a difference between Non-carrier/PMC/SMC** | **PMC vs Non-carrier** | **SMC vs Non-carrier** | **SMC vs PMC** |
| --- | --- | --- | --- | --- |
| Entorhinal | P<0.0001 | 0.01  ( -0.07, 0.08)  (P=0.87) | 0.68  ( 0.50,0.86)  (P<0.001) | 0.67  (0.48, 0.86)  (P<0.001) |
| Metatemporal | P<0.0001 | -0.003  (-0.06 , 0.05) (P=0.91) | 0.88  (0.60 , 1.16) (P<0.001) | 0.88  (0.60 , 1.17) (P<0.001) |
| Composite- global | P<0.0001 | 0.01  (-0.05 , 0.07) (P=0.79) | 0.82  (0.58 , 1.05) (P<0.001) | 0.81  (0.57 , 1.05) (P<0.001) |
| Postcingulate | P<0.0001 | 0.08  (-0.02, 0.18 ) (P=0.12) | 1.73  (1.22 , 2.23) (P<0.001) | 1.65  (1.13 , 2.16 ) (P<0.001) |
| Precuneus | P<0.0001 | 0.07  (-0.03 , 0.17) (P=0.18) | 1.66  (1.25 , 2.07 ) (P<0.001) | 1.59  (1.17 , 2.01) (P<0.001) |

Abbreviations: PMC = presymptomatic mutation carrier; SMC = symptomatic mutation carrier. *Estimated differences are reported in the style “A vs B” where, for example, the 0.67 for Entorhinal SMC vs PMC should be interpreted as the mean FTP SUVR uptake in SMC being an estimated 0.67 higher than in PMC.

**Supplementary Table 1b: Estimated covariate coefficients from the FTP SUVR group comparison models reported in Supplementary Table 1a:**

| **Entorhinal** | **Beta coefficient (95% CI) p-value** |
| --- | --- |
| **Group**   - Non-carrier - PMC - Symptomatic | -  0.01 (-0.07, 0.08) p=0.87  0.68 (0.50, 0.86) p<0.001 |
| **Study site**   - DIAN - UCL | -  -0.03 (-0.10, 0.05) p=0.51 |
| **Age** | 0.001 (-0.002, 0.005) p=0.41 |
| **Sex**   - Female - Male | -  -0.002 (-0.07, 0.07) p=0.95 |

| **Metatemporal** | **Beta coefficient (95% CI) p-value** |
| --- | --- |
| **Group**   - Non-carrier - PMC - Symptomatic | -  -0.003 (-0.06, 0.05) p=0.91  0.88 (0.60, 1.16 ) p<0.001 |
| **Study site**   - DIAN - UCL | -  -0.05 (-0.12, 0.01) p=0.08 |
| **Age** | 0.002 ( -0.001, 0.005) p=0.12 |
| **Sex**   - Female - Male | -  0.03 (-0.02, 0.09) p=0.25 |

| **Composite-global** | **Beta coefficient (95% CI) p-value** |
| --- | --- |
| **Group**   - Non-carrier - PMC - Symptomatic | -  0.01 (-0.05, 0.07) p=0.79  0.82 (0.58, 1.04) p<0.001 |
| **Study site**   - DIAN - UCL | -  -0.05 (-0.11, 0.02) p=0.16 |
| **Age** | 0.001 (-0.002, 0.004) p=0.55 |
| **Sex**   - Female - Male | -  0.03 ( -0.03, 0.09) p=0.28 |

| **Postcingulate** | **Beta coefficient (95% CI) p-value** |
| --- | --- |
| **Group**   - Non-carrier - PMC - Symptomatic | -  0.08 (-0.02, 0.19) p=0.12  1.73 (1.22, 2.23) p<0.001 |
| **Study site**   - DIAN - UCL | -  -0.02 (-0.11, 0.06) p=0.56 |
| **Age** | 0.001 (-0.002, 0.004) p=0.49 |
| **Sex**   - Female - Male | -  0.05 (-0.02, 0.13) p=0.15 |

| **Precuneus** | **Beta coefficient (95% CI) p-value** |
| --- | --- |
| **Group**   - Non-carrier - PMC - Symptomatic | -  0.07 (-0.03, 0.18) p=0.18  1.66 (1.25, 2.07) p<0.001 |
| **Study site**   - DIAN - UCL | -  -0.03 (-0.10, 0.05) p=0.46 |
| **Age** | 0.0001 (-0.003, 0.003) p=0.97 |
| **Sex**   - Female - Male | -  0.05 (-0.02, 0.12) p=0.16 |

**Supplementary Table 2a: Estimated difference* in mean rates of change of FTP SUVR uptake (SUVR/year) (and 95% CI) across regions of interest, after adjusting for age, sex and study site**

| **Region** | **PMC vs Non-carrier** |
| --- | --- |
| Entorhinal | -0.04 (-0.09, 0.01) p=0.08 |
| Meta-temporal | -0.03 (-0.07, 0.02) p=0.20 |
| Composite-global | -0.03 (-0.06, 0.01) p=0.20 |
| Postcingulate | 0.01 (-0.05, 0.07 ) p=0.74 |
| Precuneus | 0.01 (-0.04, 0.07) p=0.62 |

**Abbreviation: PMC = presymptomatic mutation carrier. *Estimated differences are reported as “PMC vs Non-carrier” where, for example, the -0.04 for Entorhinal should be interpreted as the mean rate of change of FTP SUVR uptake in PMC being an estimated (statistically not significant) 0.04 SUVR/year lower than in non-carriers.**

**Supplementary Table 2b:** **Estimated covariate coefficients from the FTP SUVR rates of change group comparison models reported in Supplementary Table 2a:**

| **Entorhinal** | **Beta coefficient (95% CI) p-value** |
| --- | --- |
| **Group**   - Non-carrier - PMC | -  -0.04 (-0.09, 0.01) p=0.08 |
| **Study site**   - DIAN - UCL | -  0.004 (-0.041, 0.048) p=0.87 |
| **Age** | -0.002 (-0.006, 0.002) p=0.25 |
| **Sex**   - Female - Male | -  -0.07 (-0.12, -0.02) p=0.01 |

| **Meta-temporal** | **Beta coefficient (95% CI) p-value** |
| --- | --- |
| **Group**   - Non-carrier - PMC | -  -0.03 (-0.07, 0.02) p=0.20 |
| **Study site**   - DIAN - UCL | -  0.004 (-0.03, 0.04) p=0.79 |
| **Age** | -0.001 (-0.005, 0.002) p=0.37 |
| **Sex**   - Female - Male | -  -0.04 (-0.08, 0.0003) p=0.05 |

| **Composite-global** | **Beta coefficient (95% CI) p-value** |
| --- | --- |
| **Group**   - Non-carrier - PMC | -  -0.03 (-0.06, 0.01) p=0.20 |
| **Study site**   - DIAN - UCL | -  -0.003 (-0.04, 0.03) p=0.85 |
| **Age** | -0.002 (-0.004, 0.001) p=0.28 |
| **Sex**   - Female - Male | -  -0.05 (-0.09, -0.01) p=0.01 |

| **Postcingulate** | **Beta coefficient (95% CI) p-value** |
| --- | --- |
| **Group**   - Non-carrier - PMC | -  0.01 (-0.05, 0.07 ) p=0.74 |
| **Study site**   - DIAN - UCL | -  0.01 (-0.05, 0.06) p=0.80 |
| **Age** | 0.00004 (-0.004, 0.005) p=0.99 |
| **Sex**   - Female - Male | -  -0.08 (-0.15, -0.01) p=0.04 |

| **Precuneus** | **Beta coefficient (95% CI) p-value** |
| --- | --- |
| **Group**   - Non-carrier - PMC | -  0.01 (-0.04, 0.07) p=0.62 |
| **Study site**   - DIAN - UCL | -  0.01 (-0.06, 0.07) p=0.86 |
| **Age** | -0.0002 (-0.005, 0.005) p=0.93 |
| **Sex**   - Female - Male | -  -0.07 (-0.14, 0.001) p=0.05 |

|  | **Tau (FTP SUVR) (MC vs NC) earliest significant (p<0.05) difference**  **(EYO)**  **(p-value**)** | **Estimated difference* (MC vs NC)**  **(FTP SUVR) (95% CI)** |
| --- | --- | --- |
| Precuneus | -10  (p=0.03) | 0.22  (0.02, 0.43) |
| Postcingulate | -7  (p=0.02) | 0.40  (0.07, 0.73) |
| Entorhinal | -7  (p= 0.04) | 0.16  (0.01, 0.31) |
| Meta-temporal | -6  (p= 0.03) | 0.20  (0.02, 0.38) |
| Composite-global | -6  (p= 0.02) | 0.20  (0.03, 0.37) |

**Supplementary Table 3a: Ordering of outcomes by estimated years to symptom onset (EYO) point at which a significant difference in estimated mean FTP uptake was identified between mutation carriers (MC) and noncarriers (NC), after adjusting for age at visit, sex and study site. *Estimated differences are reported as “MC vs NC” where, for example, the 0.22 for Precuneus should be interpreted as the mean FTP SUVR uptake in MC being an estimated 0.22 higher than in NC. ****Test of the null hypothesis that the adjusted difference is zero

**Supplementary Table 3b: Estimated covariate coefficients from from the models reported in Supplementary Table 3a, which were used to obtain the trajectories shown in Supplementary Figure 3.**

| **Entorhinal** | **Beta coefficient (95% CI) p-value** |
| --- | --- |
| **Group**   - Non-carrier - Mutation carrier | -  0.34 (0.18,0.50) p<0.001 |
| **Study site**   - DIAN - UCL | -  -0.05 (-0.20, 0.10) p=0.50 |
| **Age** | 0.01 (-0.0002, 0.02) p=0.06 |
| **Sex**   - Female - Male | -  0.08 (0.01, 0.15) p=0.03 |
| **EYO** | -0.01 (-0.02, 0.002) p=0.11 |
| **Group#EYO interaction**   - Non-carrier#EYO - Mutation carrier#EYO | -  0.03 (0.02, 0.04) p<0.001 |
| **EYO^2^ (quadratic term included only for mutation carriers)** | 0.0005 (-0.0002, 0.001) p=0.14 |

| **Metatemporal** | **Beta coefficient (95% CI) p-value** |
| --- | --- |
| **Group**   - Non-carrier - Mutation carrier | -  0.52 (0.33, 0.71) p<0.001 |
| **Study site**   - DIAN - UCL | -  -0.15 (-0.33, 0.04) p=0.12 |
| **Age** | 0.01 (0.002,0.02) p=0.03 |
| **Sex**   - Female - Male | -  0.105 (0.03, 0.16) p=0.004 |
| **EYO** | -0.01 (-0.02, 0.0003) p=0.06 |
| **Group#EYO interaction**   - Non-carrier#EYO - Mutation carrier#EYO | -  0.06 (0.05, 0.08) p<0.001 |
| **EYO^2^ (quadratic term included only for mutation carriers)** | 0.001 (0.001, 0.002) p<0.001 |

| **Composite-global** | **Beta coefficient (95% CI) p-value** |
| --- | --- |
| **Group**   - Non-carrier - Mutation carrier | -  0.52 (0.34, 0.69) p<0.001 |
| **Study site**   - DIAN - UCL | -  -0.13 (-0.301 0.04) p=0.12 |
| **Age** | 0.01 (0.001, 0.02) p=0.04 |
| **Sex**   - Female - Male | -  0.10 (0.04, 0.17) p=0.002 |
| **EYO** | -0.01 (-0.02, -0.0002) p=0.05 |
| **Group#EYO interaction**   - Non-carrier#EYO - Mutation carrier#EYO | -  0.06 (0.05, 0.08) p<0.001 |
| **EYO^2^ (quadratic term included only for mutation carriers)** | 0.001 (0.001, 0.002) p<0.001 |

| **Postcingulate** | **Beta coefficient (95% CI) p-value** |
| --- | --- |
| **Group**   - Non-carrier - Mutation carrier | -  1.07 (0.73, 1.41) p<0.001 |
| **Study site**   - DIAN - UCL | -  -0.11 (-0.44, 0.23) p=0.54 |
| **Age** | 0.02 (-0.00006, 0.04) p=0.05 |
| **Sex**   - Female - Male | -  0.13 (0.06, 0.20) p<0.001 |
| **EYO** | -0.02 (-0.04, -0.001) p=0.04 |
| **Group#EYO interaction**   - Non-carrier#EYO - Mutation carrier#EYO | -  0.11 (0.08, 0.14) p<0.001 |
| **EYO^2^ (quadratic term included only for mutation carriers)** | 0.002 (0.001, 0.003) p=0.001 |

| **Precuneus** | **Beta coefficient (95% CI) p-value** |
| --- | --- |
| **Group**   - Non-carrier - Mutation carrier | -  0.98 (0.67, 1.28) p<0.001 |
| **Study site**   - DIAN - UCL | -  -0.10 (-0.40, 0.20) p=0.51 |
| **Age** | 0.02 (-0.001, 0.03) p=0.07 |
| **Sex**   - Female - Male | -  0.11 (0.04, 0.18) p=0.004. |
| **EYO** | -0.02 (-0.03, 0.001) p=0.06 |
| **Group#EYO interaction**   - Non-carrier#EYO - Mutation carrier#EYO | -  0.10 (0.07, 0.13) p<0.001 |
| **EYO^2^ (quadratic term included only for mutation carriers)** | 0.002 (0.001, 0.003) p=0.004 |
